# Artificial intelligence-aided endoscopic in-line particle size analysis during the pellet layering process

**DOI:** 10.1016/j.jpha.2025.101227

**Published:** 2025-02-12

**Authors:** Orsolya Péterfi, Nikolett Kállai-Szabó, Kincső Renáta Demeter, Ádám Tibor Barna, István Antal, Edina Szabó, Emese Sipos, Zsombor Kristóf Nagy, Dorián László Galata

**Affiliations:** a Department of Organic Chemistry and Technology, Faculty of Chemical Technology and Biotechnology, Budapest University of Technology and Economics, Műegyetem rkp. 3., H-1111, Budapest, Hungary; b Department of Pharmaceutics, Semmelweis University, Hőgyes E. str 7, 1092, Budapest, Hungary; c Center for Pharmacology and Drug Research & Development, Semmelweis University, Budapest, Hungary; d Department of Pharmaceutical Industry and Management, Faculty of Pharmacy, George Emil Palade University of Medicine, Pharmacy, Sciences and Technology of Targu Mures, Gheorghe Marinescu Street 38, 540142, Targu Mures, Romania

**Keywords:** Machine vision, Convolutional neural networks, In-line monitoring, Endoscope, Particle size distribution, Pellet layering, Process analytical technology

## Abstract

In this study, an artificial intelligence-based machine vision system was developed for in-line particle size analysis during the pellet layering process. Drug-layered pellets were produced by coating microcrystalline cellulose cores with an ibuprofen-containing layering liquid until the target drug content was achieved. Drug content increases with pellet size; therefore, particle size monitoring can ensure product safety and quality. The direct imaging system, consisting of a rigid endoscope, a light source, and a high-speed camera, provides real-time information about pellet size and layer uniformity, enabling timely intervention in the case of out-of-spec products. A convolutional neural network-based instance segmentation algorithm was employed to detect particles in focus, ensuring that pellet size could be accurately determined despite the dense flow of the particles. After training the model, the performance of the developed system was assessed by analysing the particle size distribution of pellet cores with variable sizes within the 250–850 μm size range. The endoscopic system was tested in-line at a larger scale during the drug layering of inert pellet cores. The particle size data acquired in real time with the endoscopic imaging system corresponded with the reference methods, demonstrating the feasibility of the proposed machine vision-based method as a process analytical technology tool for in-line process monitoring.

## Highlights

• AI-aided endoscopic imaging system was developed for in-line particle size analysis.
• CNN-based instance segmentation algorithm provided detailed particle outline.
• In-time monitoring of pellet size distribution and shape during the layering process.
• Enhanced understanding of the particle size increase dynamics.

## Graphical abstract

**Figure:**
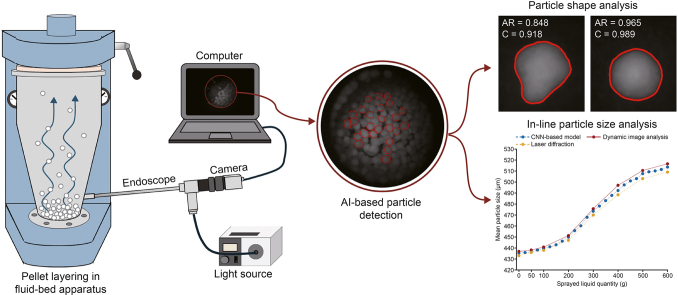

## 1 Introduction

Pellets are small free-flowing agglomerates with a near spherical or cylindrical shape, utilised across various industries due to their narrow particle size distribution and uniform shape, which provide advantages during processing and handling. Applications include animal feeds, fertilizers, catalyst carriers, iron ores, plastics and pharmaceuticals [1]. In the pharmaceutical industry, pellets are spherical particles with a diameter between 0.2 and 2.0 mm, commonly used in the production of oral dosage forms such as capsules and tablets [2]. Pellets offer several advantages, including enhanced flowability, reduced particle segregation, mitigation of excessive dust, improved distribution within the gastrointestinal tract, reduced risk of dose dumping of modified release dosage forms, and enabling the simultaneous administration of incompatible substances [3,4]. Pharmaceutical pellets are manufactured by various techniques, such as extrusion-spheronization, layering, cryopelletization, freeze pelletization, and hot-melt extrusion [5,6]. Drug-layered pellets are produced by layering of inert pellet cores with active pharmaceutical ingredient (API) until the desired dosage content is achieved. The API content in these pellets is determined by particle size, with variations in drug load corresponding to changes in layer thickness. The fluid bed apparatus is widely utilised in the pharmaceutical industry for drug layering, as it efficiently integrates multiple process steps—preheating, spraying-layering, and drying—into a single piece of equipment [1,7,8]. This technique is also suitable for coating the pellets with rate-controlling film forming polymers to achieve modified-release (MR) profiles [2,9,10].

The critical quality attributes (CQAs) of pellets—such as particle size, particle size distribution, particle shape, density, surface area, and moisture content—play a pivotal role in determining the overall performance of the final product. Variations in particle size can cause differences in the surface area available for coating, which may influence the amount of coating material applied. Consequently, a broad particle size distribution could lead to varying coating thickness, potentially affecting the drug content of drug-layered pellets and altering the drug release profile of MR formulations [[11], [12], [13], [14]]. Additionally, particle morphology plays a crucial role in the flowability of the sample; pronounced surface roughness in pellets can hinder capsule filling [15,16]. When pellets with non-uniform surfaces are coated, the rate-controlling layers may become uneven, potentially compromising drug release. Therefore, it is crucial to monitor these critical quality attributes to ensure the desired product quality.

Various critical process parameters (CPP) influence the aforementioned CQAs, including inlet air temperature, product temperature, relative humidity, spray rate, and atomization air pressure. Elevated air temperatures can cause sprayed droplets to dry too quickly, leading to uneven distribution on the pellet surface and resulting in rough, porous coatings. Conversely, low product temperatures can result in unwanted particle agglomeration because the drug layers remain wet, causing the pellets to stick together. The spray rate influences both the droplet size of the wetting liquid and its distribution uniformity within the solid mass, with higher rates resulting in larger pellets and a broader particle size distribution [[17], [18], [19], [20], [21]].

Even minor deviations in the CPPs can significantly impact product quality, potentially leading to batch rejects and recalls. Real-time monitoring of the CQAs enables the timely adjustment of the CPPs, ensuring that the CQAs remain within the specified target range. Traditionally, CQAs are assessed at the end of the fluid-bed process by taking a sample from the apparatus for off-line analysis; however, these methods are destructive, time-consuming and do not allow for real-time feedback control [22]. A recent shift in pharmaceutical manufacturing has led to the adoption of the process analytical technology (PAT) initiative, which aims to improve product safety and quality by monitoring the CQAs and CPPs with in-process sensors, coupled with the corresponding data analysis methods and control strategy [23,24]. Many PAT tools have demonstrated the capability of monitoring the pellet layering and coating process, including near-infrared (NIR) spectroscopy [[25], [26], [27]], Raman spectroscopy [28,29], spatial filter velocimetry (SFV) [[30], [31], [32]], focused beam reflectance measurement (FBRM) [33], photometric stereo imaging [34], Terahertz pulsed imaging [35] and acoustic emission spectroscopy [36]. Machine vision stands out as an extremely versatile PAT tool for real-time analysis. Digital imaging has seen a tremendous progress in the last decade, driven by the development of high frame-rate cameras capable of real-time data acquisition and the increased efficiency of graphical processing units (GPU) that reduce processing time [37]. Literature describes numerous machine vision-based methods to monitor the pellet layering and coating process. Digital imaging systems consisting of an industrial camera, light source and lens were employed for pellet size and shape analysis. Možina et al. [38] implemented a digital imaging system on-line using a vibratory feeder, which minimized particle overlap and ensured continuous particle flow. For in-line application, Oman Kadunc et al. [39] positioned a camera-based imaging system externally on the observation window of the fluid-bed apparatus. In order to extract quantitative information from the captured pictures, the authors employed image segmentation to separate the pellets from the background. The performance of the aforementioned image analysis method declines with dense material flow due to the extensive particle overlap. Furthermore, the changing image background and out-of-focus particles often make segmentation more difficult [40].

Convolutional neural networks (CNN) have revolutionized image-based object recognition. CNNs are a class of deep learning models designed to process and analyse visual data through multiple layers of convolutional operations. Object detection algorithms classify and locate objects within an image using bounding boxes, while instance segmentation outlines the exact contours of each object [41,42]. Hosseinzadeh et al. [43] developed an off-line camera-based imaging system to investigate the shape and size of iron oxide pellets, utilising a mask region-based convolutional neural network (Mask R–CNN) algorithm to detect particles. Mehle et al. [44,45] have mounted an in-line camera-based visual inspection system on the observation window of the fluid-bed apparatus and a CNN-based instance segmentation algorithm was used to detect pellet agglomeration during pharmaceutical pellet coating. However, since not all fluid-bed apparatuses are equipped with observation windows, integrating these systems across different setups remains a challenge. Endoscopes can be easily integrated and automated with existing laboratory hardware and software, and have been utilised for in-line monitoring of pharmaceutical processes. Simon et al. [46] presented the first endoscopy-based particle imaging system for the crystallization process, where video data was analysed using the mean gray intensity method and digital image processing to detect the initial formation of crystals during nucleation. Our previous work demonstrated the feasibility of an artificial intelligence (AI)-based endoscopic imaging system for the particle size measurement of granules [47]. An endoscope can be easily installed through a sampling port without requiring modifications to the apparatus. The imaging system was tested under laboratory conditions using a three-dimensional (3D)-printed device that simulated particle flow during fluidization. Bounding box-based object detection was utilised to identify the granules in focus, which limited the system's ability to investigate particle morphology.

In the current study, a machine vision based PAT system was developed for the in-line monitoring of a pellet layering process. A convolutional neural network-based instance segmentation algorithm was employed to detect the outline of pellets, allowing a more precise particle size measurement and accurate determination of particle shape. The endoscopic system was integrated in-line into a fluid-bed apparatus through the sampling port with no modification of the equipment. This application marks a significant scale-up from our previous proof-of-concept experiments, where testing was conducted during particle fluidization in a small 3D printed apparatus. In the current experiments, the drug containing coating liquid was sprayed on the pellets, thus the particle size and drug content increased throughout the pellet layering process. The performance of the AI-based technique was evaluated by comparing the particle size obtained in real-time with off-line reference measurements. Additionally, the dynamics of particle growth during the pellet layering process were assessed. To the authors' knowledge, this is the first in-line particle size and shape measurement method employing an artificial intelligence-based endoscopic system during the pellet layering process.

## 2 Materials and methods

### 2.1 Materials

Sugar spheres (various sizes within the 250–850 μm range, Pharm-a-Spheres®, H.G.Werner GmbH, Kiel, Germany) and microcrystalline cellulose (MCC) spheres (355–500 μm; Ethispheres® 850, NPPharm Ltd., Bazainville, France) were used as inert cores. Hydroxypropyl methylcellulose (HPMC; Pharmacoat 606, Shin-Etsu Chemical Ltd., Tokyo, Japan) was used as binder for the drug-layering process. Ibuprofen sodium (Sigma-Aldrich Chemie GmbH, Steinheim, Germany) was used as model drug.

### 2.2 Methods

#### 2.2.1 Pellet layering

Ibuprofen sodium loaded pellets were prepared by layering the drug-binder liquid onto the inert cores using a bottom-spraying technique (nozzle diameter: 0.8 mm). A batch of 200 g MCC pellet cores was layered in a laboratory-scale fluid bed dryer Aeromatic Strea-I (Aeromatic Fielder AG, Bubendorf, Switzerland). The 600 g of aqueous layering liquid contained 10 g HPMC and 25 g ibuprofen sodium. The inlet air flow rate was set to 100 m³/h, the inlet air temperature was maintained at 50 °C, and the outlet air temperature was kept at 40 ± 3 °C. The atomizing pressure was 1 bar, and the spray rate was controlled at 2.0 g/min. After every 100 g of the layering liquid was sprayed, the batch was dried for 5 min. At the end of the layering process, the pellets were dried until the outlet air temperature reached the inlet air temperature value of 45 °C. Samples for drug content measurement and off-line particle size analysis were collected at various stages of the layering process (at 50, 100, 200, 300, 400, 500, 600 g of layering solution added).

#### 2.2.2 Image acquisition setup

The direct imaging system consisted of a rigid endoscope, a light source and a process camera, enabling real-time monitoring of the particles. The camera was operated with a 900 × 900 pixels resolution at 50 frames per second (fps) and a shutter speed of 75 μs. The pixel-to-μm ratio of the imaging system was determined using a caliper. Image analysis was carried out using AI-based image analysis software (Videometry 1.2.2, QDevelopment, Budapest, Hungary). The software employs a CNN model to detect and outline particles in the images. The equivalent circular diameter of the particles was initially calculated in pixels and subsequently converted to micrometers. Real-time image analysis was performed on a laptop equipped with an Nvidia GTX 1060 GPU (NVIDIA Corporation, Santa Clara, CA, US) and an Intel i7 7700 HQ CPU (Intel Corporation, Santa Clara, CA, USA), ensuring that the software kept pace with the 50 fps image acquisition rate.

The endoscopic system was first tested with a custom-made 3D-printed device designed to simulate fluidization in a fluid-bed apparatus using pressurized air (Fig. S1). The sugar pellet cores were placed in the 3D-printed device and videos were recorded during fluidization. Individual video frames were extracted to create the dataset containing examples of the particles. The training dataset was created by annotating the pellets in focus to ensure that the CNN detects only those whose size can be precisely determined. The training dataset consisted of 500 images with a total of 1,768 annotations of the pellets. The dataset contained 100 images from each of the five different sugar starter pellet fractions within the 250–850 μm range (250–355, 355–500, 500–600, 600–710, 710–850 μm). Manual annotation of the images was carried out using the smart polygon function in the Roboflow online image annotation tool [48], enabling quicker annotation compared to fully manual methods. This approach utilises a machine learning model to generate suggestions for particle outlines; however, manual correction of the outlines was still necessary. Basic image augmentation techniques—rotation and flipping—were used to artificially expand the size of the training dataset by 4 times. The final dataset was split into training (70%), validation (20%) and test (10%) sets. The model was trained for 100 epochs with a batch size of 10 and the input image size was set to 928 by 928 pixels. The final model architecture comprised 165 layers with a total of 7,398,422 parameters. Performance metrics, including mean average precision (mAP), precision (P), and recall (R), were utilised to evaluate the effectiveness and accuracy of the trained model. Precision quantifies the accuracy of the model's positive predictions, representing the ratio of true positives to all predicted positives. Recall measures the model's ability to identify all relevant instances, calculated as the ratio of true positives to all actual positives. The F1-score is the harmonic mean of precision and recall, providing a balanced measure of model performance by accounting for both false positives and false negatives. mAP provides an overall performance measure by computing the area under the precision-recall curve. The best model had a *P* value of 0.689, R value of 0.791, and F1-score of 0.736. The mAP-50 value was 0.781, while mAP50-95 was 0.671.

After training, the model was used to perform image analysis on additional videos that were not included in the training data. The suitability of the imaging system was evaluated by determining the particle size distribution (PSD) of various spherical sugar starter pellet fractions within the 250–850 μm range.

**Fig. 1:**
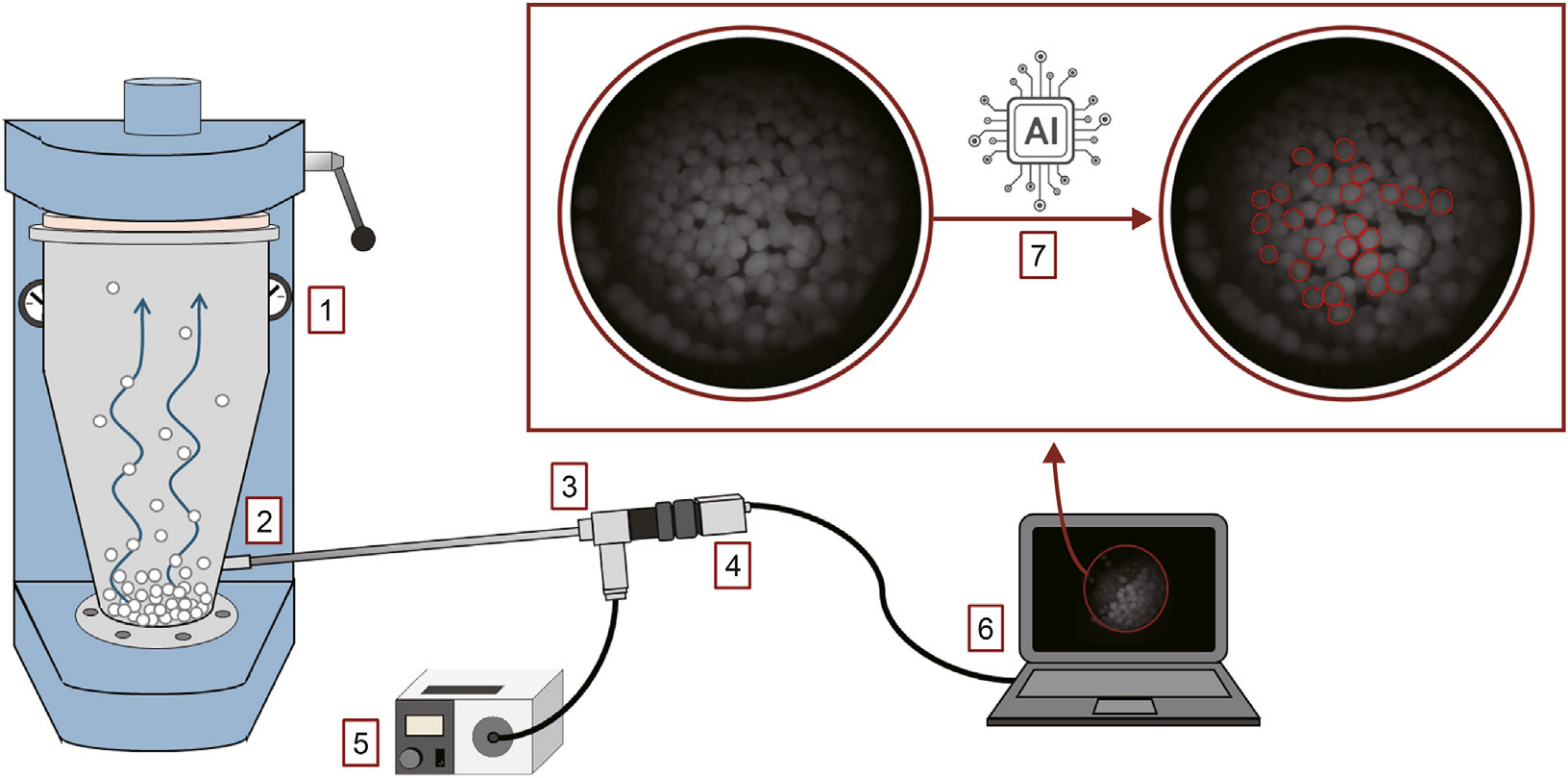
Experimental setup for in-line particle size analysis: (1) fluid-bed apparatus, (2) sampling port, (3) rigid endoscope, (4) camera, (5) light source, (6) computer, and (7) convolutional neural networks (CNN)-based software. AI: artificial intelligence.

The developed image analysis system was tested in-line during the drug layering of MCC pellet cores. Fig. 1 shows the schematic drawing of the experimental setup for the in-line imaging system. The endoscope was inserted through the sampling port of the fluid-bed apparatus.

#### 2.2.3 Off-line measurement of pellet size

Two off-line reference methods were used to determine the particle size of the samples collected at various stages of the layering process. Pellet size was measured by laser diffraction, using a Malvern Mastersizer 2000 (Malvern Instruments, Worcestershire, UK). 1 g of sample was fed into the device using the Scirocco 2000 dry sample dispersion unit (Malvern Instruments). Measurements were conducted with 0.5 bar dispersing air pressure and 6 s measurement time.

Off-line dynamic image analysis (DIA), utilising a custom software previously presented by Madarász et al. [49], served as a camera-based reference method. This is a similar arrangement to the Retsch Camsizer® (Retsch Technology GmbH, Haan, Germany) particle size and shape analyser. A vibratory feeder, fitted with a U-shaped chute, was employed to feed the samples into the measurement equipment. A Basler camera (Basler acA4112–30uc, Basler AG, Ahrensburg, Germany) was operated with a 1000 × 1000 resolution at 50 frames per second (fps) and a shutter speed of 150 μs. Positioned opposite a custom panel light (Apokromat Ltd., Budapest, Hungary), the camera captured the silhouettes of the particles in free fall. The regionprops function in MATLAB 8.2. (MathWorks, Natick, MA, USA) was used to determine various particle properties such as equivalent circular diameter, area and perimeter for every object.

The equivalent spherical diameter of the particles was measured with both reference methods and the results were expressed in volume-based distribution.

The Kolmogorov–Smirnov test was used to compare the particle size distributions, with the null hypothesis stating that the reference measurement and the endoscopic measurement were drawn from the same distribution. The null hypothesis was either rejected or accepted at a 5% significance level.

#### 2.2.4 Particle shape analysis

**Fig. 2:**
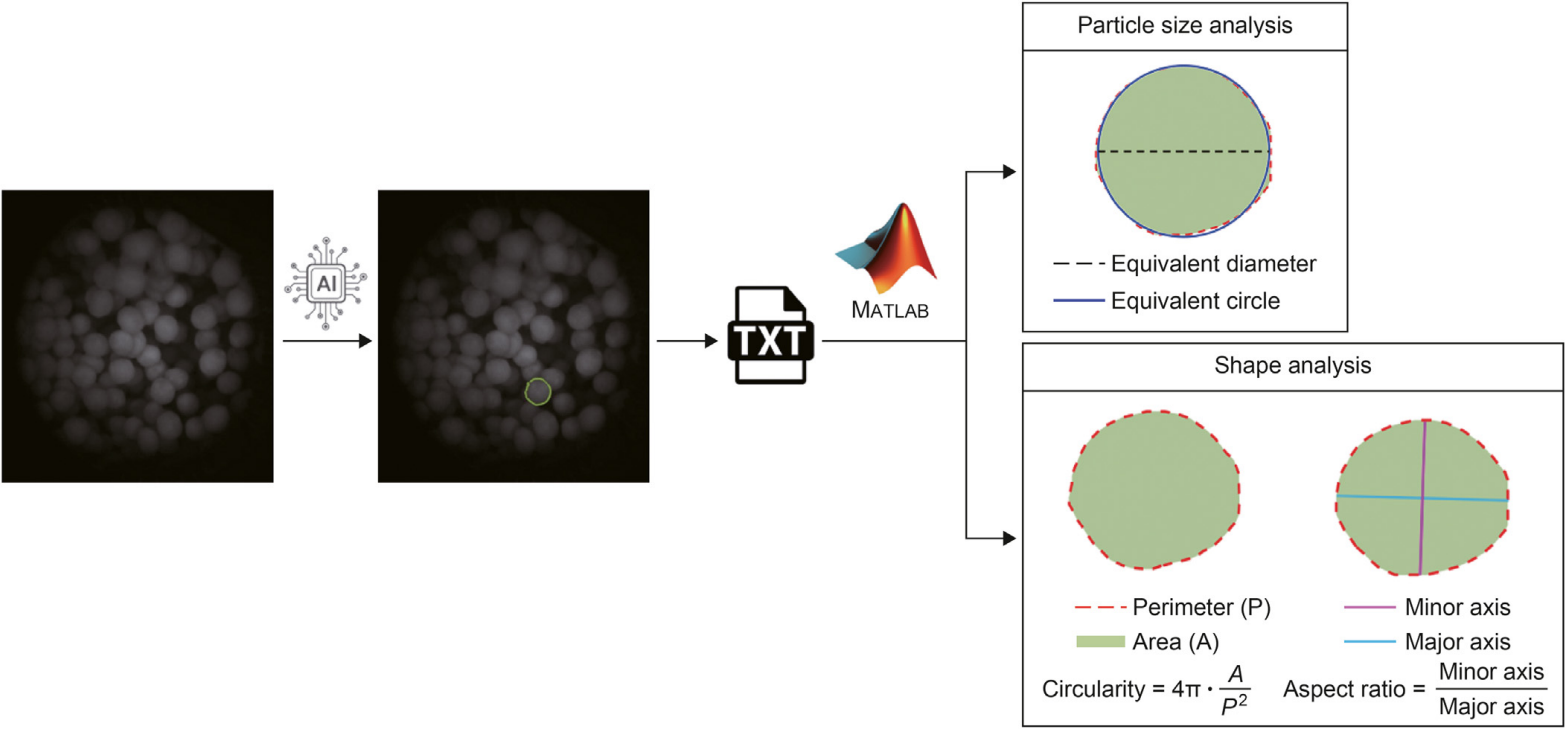
The main steps of particle size and shape analysis with the convolutional neural networks (CNN)-based imaging system: The original endoscopic images underwent artificial intelligence (AI)-based particle detection and the results were stored in a text format, which was processed in MATLAB to determine the particle size (equivalent circular diameter) and shape parameters (circularity, aspect ratio) of the pellets.

The primary steps of pellet shape analysis are illustrated in Fig. 2. The shape parameters (circularity and aspect ratio) of the pellets were determined using the outline of the AI-based detections. The output of the trained AI model was a text file containing the predicted outlines of the particles. These coordinates were processed off-line in MATLAB 8.2. with a custom-made algorithm using the regionprops function to determine the properties of each contiguous region that is 8-connected.

The circularity of the particles was calculated using the following formula:

$$C=4\pi \cdot \frac{A}{P^{2}}$$

where *A* and *P* are the area and perimeter determined with the measurement method. Aspect ratio was calculated as the ratio of the minor and major axes of the least-squares fitting ellipse of the particle contour.

Optical microscopy and off-line dynamic image analysis served as reference methods for shape analysis. 50 pellets were selected for off-line shape analysis, captured with an optical microscope (Olympus CKX53, Olympus, Tokyo, Japan) and their shape parameters were determined using image processing. The main steps are presented in the supplementary material (Fig. S2). Subsequently, the same pellets were carefully placed under the endoscope, ensuring that the particles remained unmoved to consistently capture the same side of each pellet with the endoscopic imaging system. The setup for off-line single-pellet analysis with the endoscopic method is described in Fig. S1A.

The detections of the CNN-based imaging system were also used to determine the shape of the pellets during pellet layering. Particle shape was determined off-line after the layering process by analysing the coordinates from the text files generated during in-line monitoring as shape analysis was not integrated into the Videometry software. The shape parameters of the samples collected during the layering process were analysed using off-line dynamic image analysis, previously described in Section 2.2.3.

#### 2.2.5 Drug concentration analysis

The drug content of the drug-layered pellets was analysed spectrophotometrically using an Agilent 8453 UV/VIS spectrometer (Agilent, Hewlett-Packard, CA, USA). Three parallel measurements were performed for each sample. Drug-layered pellet samples were accurately weighed (approx. 100 mg) and the dissolved the layering coat was dissolved in a volumetric flask with distilled water. Before measurement, the sample was filtered through a 1.2 μm filter (FilterBio® GF Syringe Filter, Labex Ltd., Budapest, Hungary) with the first 5 mL being discarded. Ibuprofen concentrations were measured at 222 nm in a 10 mm cuvette.

#### 2.2.6 Scanning electron microscopy

The surface roughness of the pellets was investigated by a JEOL JSM 6380LA type scanning electron microscope (JEOL, Tokyo, Japan). Samples were fixed with a conductive double-sided carbon adhesive tape. To prevent electrostatic charging, the samples were coated with a thin layer of gold prior to measurement. The scanning electron microscopy (SEM) imaging was performed in a high vacuum, with an accelerating voltage of 10 kV and a working distance of 10–15 mm.

#### 2.2.7 Surface texture analysis

The gray level co-occurrence matrix (GLCM) was used for analysing the texture of the SEM images, providing quantitative texture information such as contrast, correlation, energy, and homogeneity. Image processing was carried out in MATLAB 8.2.. The GLCM was calculated for each image using the graycomatrix function in MATLAB, which evaluates the spatial relationship between pixel intensities. Subsequently, the graycoprops function was used to extract the texture features from the GLCM. Contrast measures the intensity difference between the reference and neighbouring pixels. Correlation assesses the linear relationship between pixel pairs, indicating how closely related a pixel is to its neighbours in terms of intensity. Energy is defined as the sum of squared elements in the GLCM, and reflects the uniformity of the image texture. Homogeneity measures the closeness of the distribution of elements in the GLCM to its diagonal, and quantifies how much the texture contains similar intensity pairs. The mean texture value of three pellets was averaged at each reference point. Smoother surfaces typically exhibit lower contrast, greater energy and homogeneity, while correlation is typically higher, indicating a more predictable texture.

## 3 Results and discussion

### 3.1 Off-line application of the imaging system

**Fig. 3:**
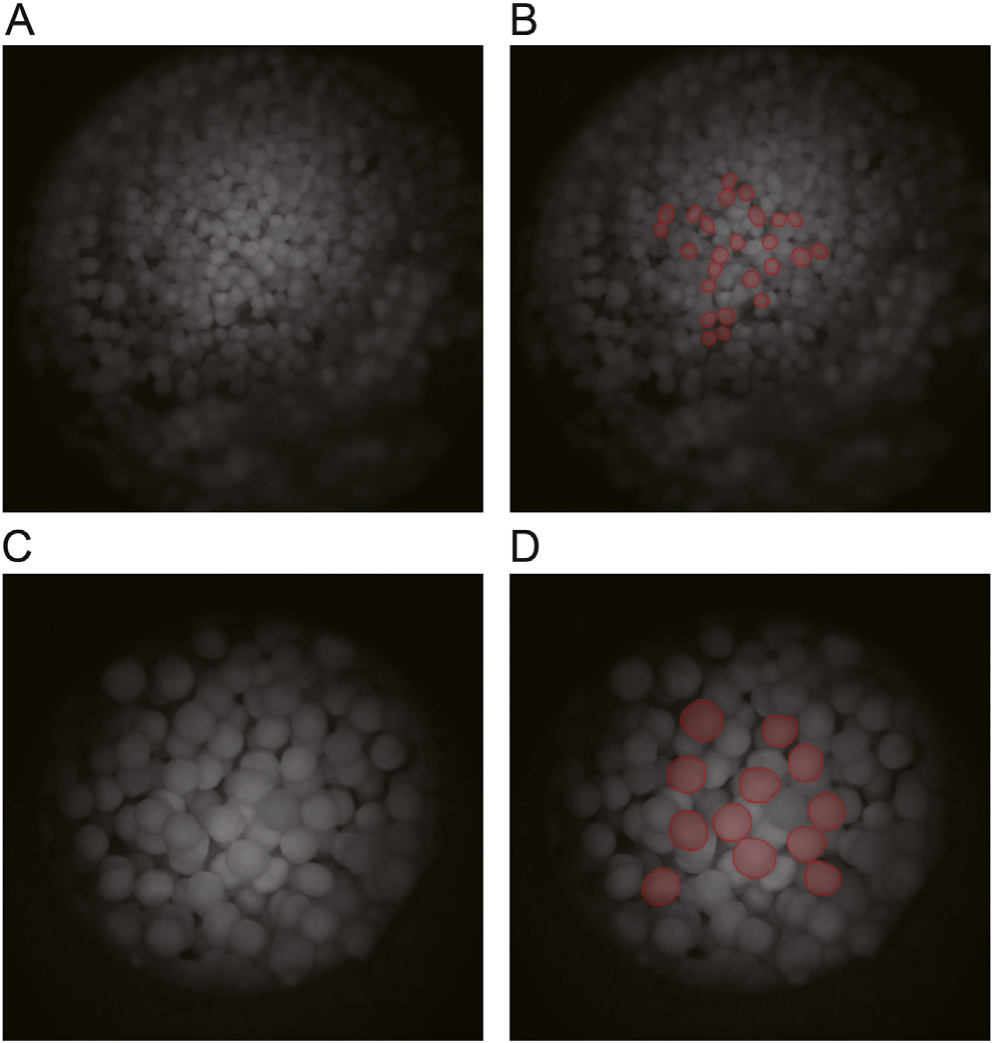
Particle detection using the trained CNN model for sugar pellet cores captured in the three-dimensional (3D)-printed fluid device. (A, B) 250–355 μm (A), and detected particles highlighted in red (B). (C, D) 710–850 μm (C) and detected particles highlighted in red (D).

The imaging system was initially tested in a series of off-line experiments to demonstrate its feasibility of in-line particle size analysis. The algorithm was trained to detect the pellets that were in focus and had a fully visible outline. Fig. 3 shows particle detection for the two pellet sizes in the images captured within the 3D-printed device. Partially visible particles were not annotated in the training dataset, and therefore, the AI model also excluded them from the detections. Additionally, the model was able to distinguish between pellets that were in focus and those which had blurry edges. The AI model assigns a confidence value to each detected object. Particles with a confidence level below 45% were excluded from the particle size analysis. This threshold was established based on visual inspection of the analysed images. The trained AI model tested on new images, obtained during off-line measurements, were not included in the training dataset. The AI-based detections were compared with an independent hand-annotated dataset, which included 25 images of the 250–355 μm pellets with 551 annotated particles and 25 images of the 710–850 μm pellets with 268 annotations, while the imaging system found 533 and 254 particles, respectively.

Particle size was determined using the predicted outlines generated by the trained AI model. The size data of 5,000 particles was used to determine the volumetric PSDs of the samples. The results were obtained by analysing a few hundred frames, which were collected in less than 15 s at a frame rate of 50 fps, capturing around 10–15 pellets per frame. This demonstrates the capability of the developed method to monitor the pellet layering process in real-time, as data acquisition takes only a few seconds, while changes in particle size occur over several minutes. The imaging system was also capable of operating at a speed of 50 fps, which included the recording of the image, applying the object detection and the extraction of particle size data from the output of the object detection algorithm.

**Fig. 4:**
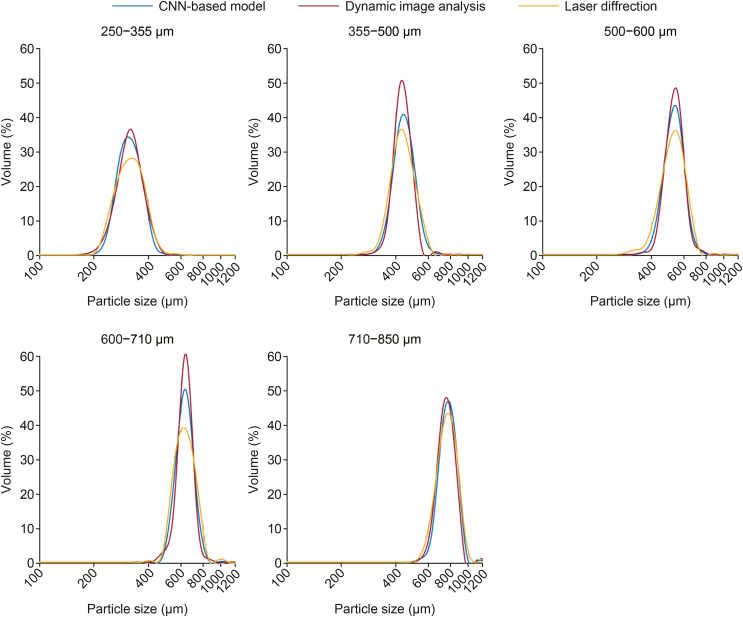
The particle size distributions of the inert pellet cores measured with the developed endoscopic system, off-line dynamic image analysis and laser diffraction. CNN: convolutional neural networks.

The PSDs obtained with the endoscopic imaging tool, as well as the two off-line reference methods (off-line dynamic image analysis and laser diffraction) are presented in Fig. 4. The *P*-values obtained from the two-sample Kolmogorov–Smirnov tests comparing the PSDs measured with the different methods are presented in Table 1. The *P*-values from the two-sample Kolmogorov–Smirnov test show that the null hypothesis (H_0_) was not rejected for any of the groups, suggesting that the particle size distributions obtained from the different measurement methods are similar.

**Table 1:** Two-sample Kolmogorov–Smirnov test results for comparing particle size measurement methods.

Sample	*P*-values from the two-sample Kolmogorov–Smirnov test comparing	
	The AI-based endoscopic system and dynamic image analysis	The AI-based endoscopic system and laser diffraction
250–355 μm	0.8899	0.9123
355–500 μm	0.8575	0.9290
500–600 μm	0.8227	0.9548
600–710 μm	0.8827	0.8827
710–800 μm	0.4889	0.9235

AI: artificial intelligence.

The average diameter (D_10_, D_50_, D_90_) and the span values are summarized in Table 2. Both the PSDs and the average diameters demonstrate a good correlation between the endoscopic imaging system and the reference methods, indicating that the CNN-based system can accurately measure the particle size of sugar pellet cores. Despite the challenges posed during particle fluidization, including dense particle flow, overlapping particles and out-of-focus particles, the results obtained with the imaging system were consistent with those obtained from off-line methods. This demonstrates the robustness of the system in collecting accurate particle data even under dynamic and challenging conditions.

**Table 2:** The D_10_, D_50_, D_90_ and span values of the inert sugar pellet cores measured with the three methods: convolutional neural networks (CNN)-based imaging system (CNN), dynamic image analysis (DIA), and laser diffraction (LD).

Sample	Method	D_10_ (μm)	D_50_ (μm)	D_90_ (μm)	Span
250–355 μm	CNN	260.38	314.78	382.87	0.39
	DIA	255.68	317.20	388.34	0.42
	LD	249.23	317.49	398.08	0.47
355–500 μm	CNN	369.52	442.73	528.57	0.36
	DIA	374.31	433.21	501.46	0.29
	LD	356.55	435.36	537.05	0.41
500–600 μm	CNN	449.83	532.12	631.41	0.34
	DIA	458.01	536.61	627.57	0.32
	LD	419.25	527.70	640.12	0.42
600–710 μm	CNN	539.65	632.58	740.85	0.32
	DIA	546.97	635.05	732.59	0.29
	LD	531.43	631.09	753.08	0.35
710–850 μm	CNN	677.03	773.47	883.49	0.27
	DIA	647.59	753.65	866.57	0.29
	LD	635.42	766.02	889.56	0.33

The results of the model can be affected by false negatives and false positives, as these represent incorrect detections that may influence the overall accuracy of the analysis. False negatives occur when particles that meet the detection criteria are not detected by the model. These errors may arise from particles that are sufficiently in focus but are incorrectly classified as out-of-focus or from particles with features that significantly differ from those in the training dataset, such as being substantially smaller or larger than the typical sizes used during model training. Undetected smaller particles can lead to a bias in the particle size distribution, shifting the results toward larger sizes. Furthermore, achieving the target sample size of 5,000 particles per data point may require additional image sampling, thereby increasing data collection time and computational effort. False positives, on the other hand, occur when the model erroneously identifies detects objects as particles in focus. These errors commonly involve partially visible particles, such as those overlapping with other particles, or particles farther from the imaging system that appear blurred. Such incorrect detections can introduce bias into the particle size distribution, potentially skewing it toward smaller sizes. The challenges associated with false negatives and false positives emphasize the necessity of a well-curated and representative training dataset. Nevertheless, the results indicate that the measurements obtained using the endoscopic imaging system closely corresponded to those from the reference methods, with minimal impact from detection errors on the overall outcomes.

**Fig. 5:**
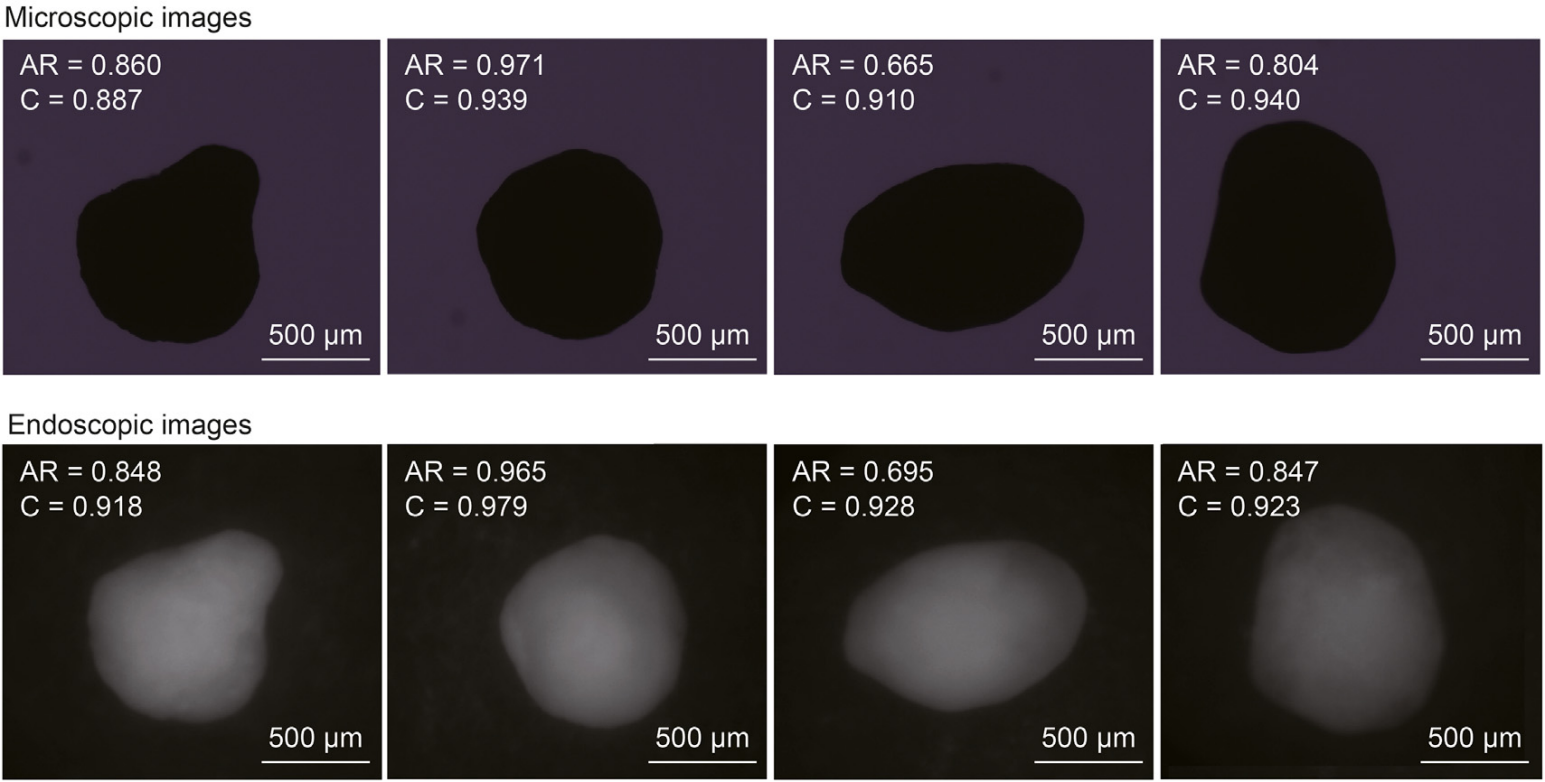
Comparison of four example particles captured with a microscope and the endoscopic imaging system. AR: aspect ratio; C: circularity.

**Fig. 6:**
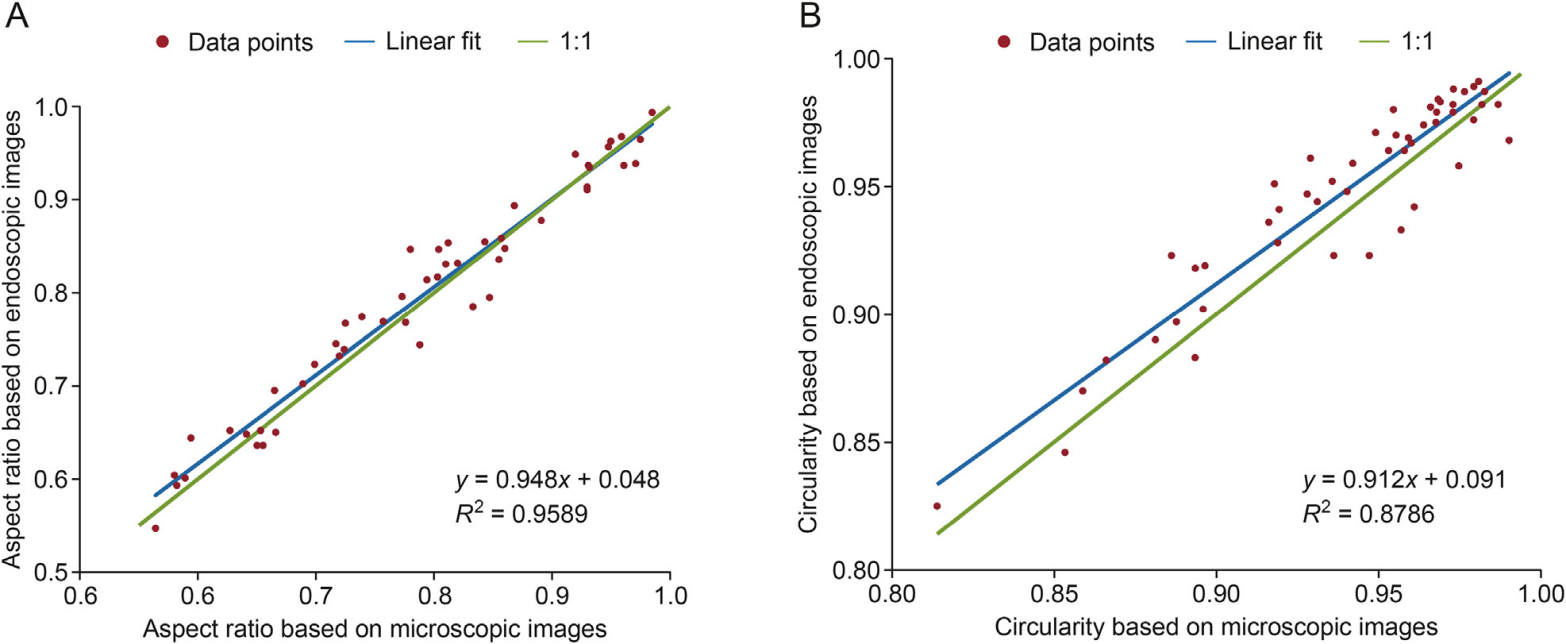
Scatter plot of the shape values of 50 pellets measured with the microscopic method and the artificial intelligence (AI)-based endoscopic imaging system. (A) Aspect ratio, and (B) circularity.

The applicability of the endoscopic imaging system for shape analysis was tested off-line by analysing the microscopic images of the same pellets. Pellets of various shapes were selected for this analysis, as presented in Fig. 5. The particle sizes determined using a CNN-based model with endoscopic images were comparable to those obtained with the reference method, which involved analysing microscopic images via image segmentation. The comparison plot of the two methods is shown in Fig. 6, which illustrates the aspect ratio and circularity value for each analysed pellet. The aspect ratios determined with the two methods were similar, with a coefficient of determination of 0.9589. Although the coefficient of determination for circularity was lower (*R*^2^ = 0.8786), this difference can be attributed to variations in outline detection between the two imaging methods. Circularity depends greatly on the particle perimeter. The resolution of the perimeter was slightly lower in the case of the CNN-based algorithm. This may be attributed to the training dataset having less detailed outlines since the pellets were manually annotated, and it was not feasible to outline them pixel by pixel. Nevertheless, the obtained results were still comparable to the reference method.

### 3.2 In-line application of the imaging system

After the particle size analysis of the various pellet fractions during fluidization in the 3D-printed device, the developed imaging system and particle recognition model were tested in-line during the pellet layering of MCC pellet cores. The location of the endoscope within the fluid-bed apparatus can affect the measurements, as lighter particles tend to fly to the upper part of the fluidized bed, while heavier and larger particles remain closer to the bottom. However, the endoscope was introduced through the system's designated sampling hole, ensuring that the measurements are representative of the particles typically sampled from this area during standard operation.

The trained machine learning model successfully detected the MCC pellets despite the training dataset only comprising images of sugar pellet cores, demonstrating the model's ability to generalize effectively and accurately identify similar objects in new, previously unseen input data. In our study, consistent lighting settings were used throughout the experiments. While variations in the reflective properties of different materials can cause some particles to appear brighter or darker in the captured images; this can be addressed by adjusting imaging parameters such as gain or gamma to align with the appearance of the training data, ensuring optimal conditions for accurate particle detection. However, despite using different materials for training (sugar pellets) and testing (MCC pellets), these adjustments were not necessary, as the particles looked similar on the captured images (Fig. S3). Flow density can also affect particle detection, and higher material flow results in increased light reflection, making particles appear brighter and, consequently, improving particle detection. Conversely, when flow density is low or fewer particles are in focus, more frames are required to collect sufficient particle size data, which increases the time needed for analysis.

**Fig. 7:**
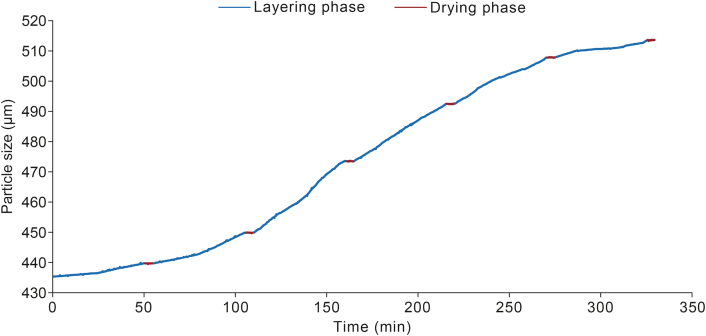
Mean particle size (D_50_) measured in real-time with the artificial intelligence (AI)-based endoscopic imaging system.

Fig. 7 illustrates the mean particle size (D_50_) over time during the pellet layering process. After every 100 g of coating liquid was sprayed, the pellets were dried for 5 min to prevent the formation of agglomerates. In the figure, the drying phases are clearly distinguished from the coating phases. The particle size increased progressively throughout the process, reflecting the consistent accumulation of the coating material on the pellet surfaces.

**Fig. 8:**
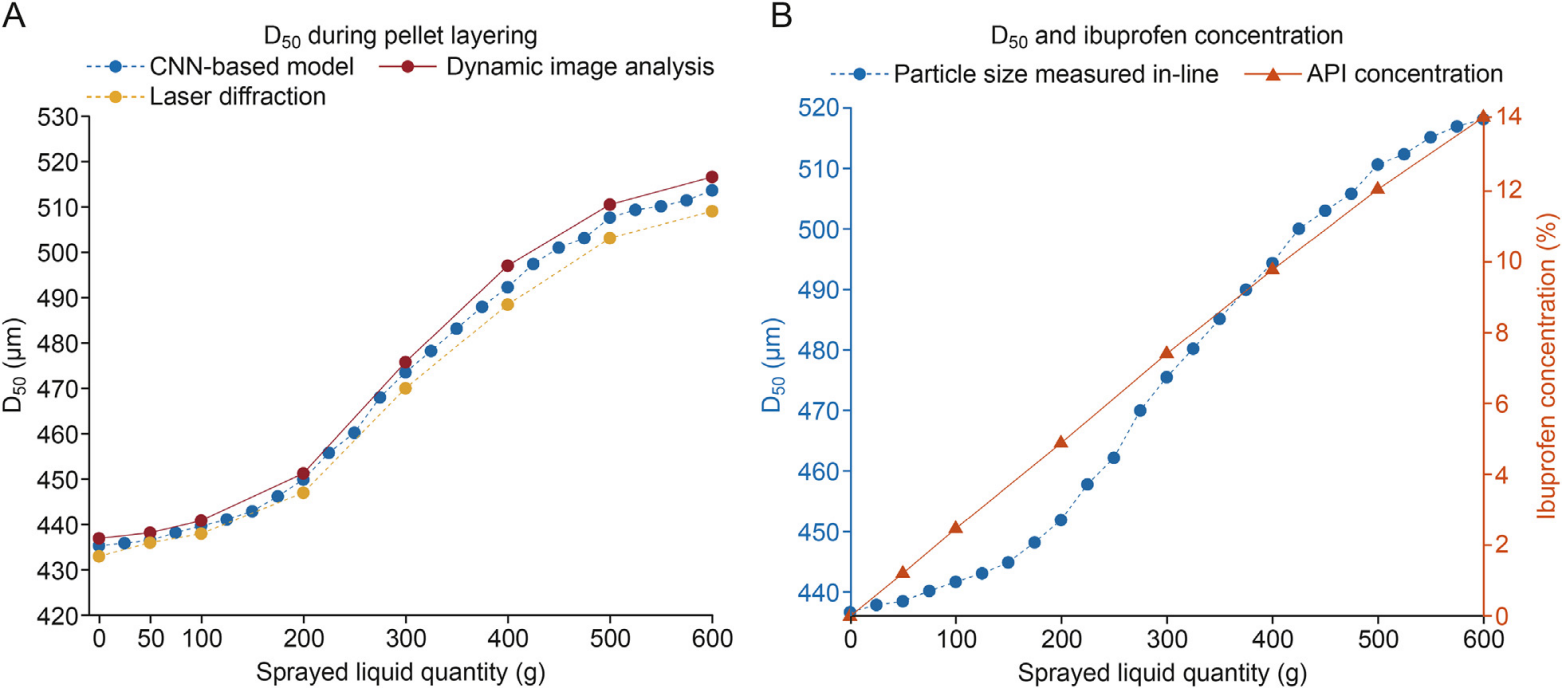
Mean particle size (D_50_) measured in-line during the pellet layering process with the developed endoscopic imaging system. The in-line particle size data is compared to (A) reference measurements and (B) the ibuprofen concentration of the pellets. CNN: convolutional neural networks; API: active pharmaceutical ingredient.

Reference samples collected at various stages of the pellet layering process were analysed with off-line reference measurements (dynamic image analysis, laser diffraction), and the results are shown in Fig. 8A. The particle size data is presented in relation to the increasing amount of drug loaded coating liquid sprayed onto the fluid bed. As the binder solution was gradually added, the mean particle size increased, reflecting the gradual accumulation of the layering solution on the particles. Particle growth was slower during the initial phase of the process, which was then succeeded by an accelerated particle growth, indicating a notable shift in the dynamics of the layering process. The in-line measurements and off-line methods showed good agreement, demonstrating the applicability of the endoscopic imaging system for in-line particle size analysis.

The shape parameters of the pellets were also monitored throughout the layering process. The mean aspect ratio and circularity, measured using both the AI-based endoscopic system and off-line dynamic image analysis, were compared at the reference points (Table S1). Initially, the mean aspect ratio of the inert pellet cores was 0.870 ± 0.070 with dynamic image analysis and 0.851 ± 0.080 with the CNN-based model. At the end of the layering process, the aspect ratio of the pellets was measured to be 0.880 ± 0.069 and 0.858 ± 0.078, respectively. The mean circularity of the pellets started at 0.956 ± 0.031 with dynamic image analysis and 0.962 ± 0.026 with the endoscopic system. At the end of the process, it was measured to be 0.964 ± 0.027 and 0.967 ± 0.025, respectively. The results obtained with the AI-based system were comparable to those from dynamic image analysis. Particle shape parameters did not significantly change over the pellet layering process. The circularity values approaching 1 indicate that the pellets have a shape close to that of a perfect circle, reflecting minimal irregularity in the perimeter relative to an equivalent circle. Meanwhile, the high aspect ratio values show minimal variation in the width-to-length ratio of the pellets.

The performance of the AI-based endoscopic system in particle size measurement is comparable to other previously established techniques. Wiegel et al. [31] utilised SFV to measure coating thicknesses up to 200 μm, achieving D_50_ measurements with a relative error below 3%, while also successfully detecting agglomerates. Hudovornik et al. [26] monitored processes where coatings of up to 80 μm thickness were applied; the D_50_ values obtained from SFV showed an *R*^2^ of 0.9933 when compared to the static image analysis reference measurement. To summarize, SFV was used to measure size increases of similar magnitude and the accuracy was on par with our method. The capability of SFV was demonstrated in the detection of agglomeration and in the measurement of size decrease due to attrition. While our current imaging setup did not include agglomeration detection, visual examination of the images obtained during monitoring revealed no evidence of such defects. Nevertheless, the potential of CNNs to detect agglomerates has been demonstrated in previous studies [44]. This capability could be integrated into the current system by training the CNN with a new detection class for agglomerates, enabling the identification of these defects during real-time monitoring. A great advantage of our AI-based method compared to SFV is that it also yields information about the shape of the particles. SFV is capable of handling various materials such as granules and pellets without prior training, while the AI system requires CNN training when particles differ significantly in appearance. A possible drawback is that the AI particle recognition software initially requires a training dataset, while SFV can yield particle size information out of the box. However, our results have shown that the object detection model, despite being trained on one type of pellet, was able to accurately recognize pellets of different compositions.

NIR spectroscopy offers another viable method for in-line monitoring of coating thickness. NIR spectroscopy demonstrated high accuracy in predicting coating thicknesses, with *R*^2^ values of 0.9961 and 0.9984 in the 0–80 μm range [26]. However, it relies on chemometric models that require recalibration for each new product, as the prediction is based on the relative intensity of signals tied to the coating composition. In contrast, the AI-based system directly measures particle size and shape, requiring only a single calibration, even when switching between different pellet compositions. However, the sensitivity of NIR spectroscopy to composition enables its application for the monitoring of other important quality attributes such as API concentration or moisture content. The equipment needed for the endoscopic system is also more affordable and easier to maintain than a NIR spectrometer.

Machine vision-based size measurement techniques have also been applied in processes involving pellets. Treffer et al. [34] employed the Eyecon™ device to capture in-line images during pellet manufacturing, achieving deviations of less than 10% compared to off-line reference measurements in most cases. Similarly, Oman Kadunc et al. [39] monitored Wurster pellet coating using a camera positioned at an observation window, measuring coating thicknesses up to 16 μm. In both of these works, the images were relatively ideal for analysis, because either all particles were in the same plane, or the particle density was low, and overlaps did not occur. Compared to these, our AI-based method can be used under more challenging imaging conditions, where the dense particle flow causes constant overlaps, and out-of-focus particles are abundant. Additionally, the endoscopic technique enables the insertion of the probe directly into industrial pellet processing equipment through sampling ports, allowing the system to be used without modifications to existing manufacturing equipment. This setup facilitates real-time particle size measurement inside a pellet coater, utilising simpler and more cost-efficient hardware compared to previous solutions.

During the pellet layering process, the layering liquid contained ibuprofen sodium as a model API, therefore with the increase of particle size, the API content of the pellets also increased. The ibuprofen concentration of the reference samples was determined and presented along the in-line particle size data acquired with the CNN-based imaging system (Fig. 8B). The API concentration demonstrated a linear increase, whereas particle growth followed an S-shaped curve. This behaviour contrasts with the expected trend, where particle growth typically starts at a faster rate and slows as the particles become larger due to reduced surface area for accumulation. To further investigate this particle growth pattern and gain a deeper understanding of the particle growth mechanism, SEM images of the reference sample surfaces were collected and analysed.

**Fig. 9:**
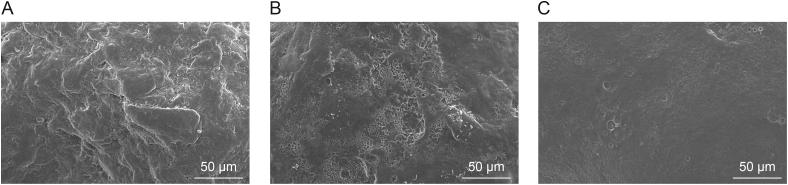
Scanning electron microscopy (SEM) images of the pellet surfaces (A) at the beginning of the layering process, (B) after spraying 100 g of binder solution, and (C) at the end of the layering process.

**Fig. 10:**
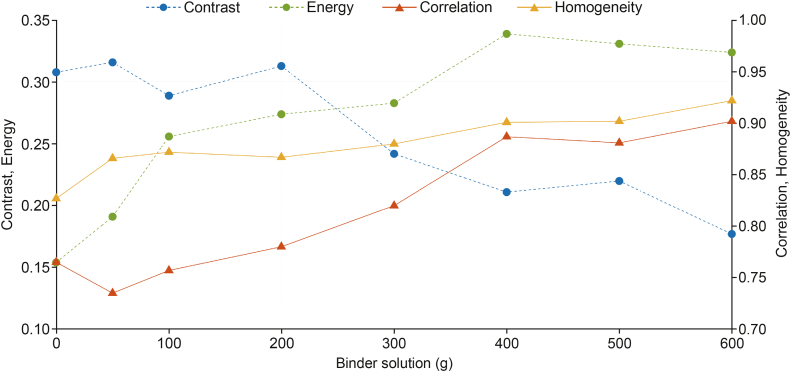
Gray level co-occurrence matrix (GLCM)-based texture features (contrast, energy, correlation, homogeneity) based on the scanning electron microscopy (SEM) images of the pellets collected during the layering process.

Fig. 9 shows the SEM images of the pellets obtained at different stages of the layering process. The surface texture parameters obtained from the GLCM analysis of the SEM images are summarized in Fig. 10. Initially, the inert pellet cores exhibited rough and porous surfaces, reflected by the lower energy and homogeneity values. Rough surfaces display abrupt changes and variations in pixel intensities and have less predictable pixel intensities, resulting in high contrast and low correlation. After starting the layering process, the correlation and contrast values initially decreased, while the homogeneity and energy values increased. Higher homogeneity and energy indicate a locally smooth and uniform texture. Conversely, the lower correlation reflects that the overall texture is more random or less consistent, with less predictable relationships between pixel values. This could be attributed to a texture with local uniformity but global variation, as initially, the sprayed droplets only partially covered the pellet surface, leaving some areas of the rough, uncoated pellet surface exposed. The exposed regions retained their original texture characteristics, reducing the linear predictability of pixel values, thus contributing to the lower correlation.

As more drug-binder liquid was sprayed onto the batch, the surface of the pellets became more uniform as shown by the greater energy and homogeneity values. The low contrast value indicates an even surface with minimal abrupt changes in pixel values. Additionally, the high correlation suggests that pixel values are strongly related, meaning that similar textures or patterns are consistently repeated, contributing to a more uniform surface.

This phenomenon offers insight into the gradual increase in pellet size observed during the initial stages of the process. The slow initial particle size increase may be linked to the wetting of the MCC pellet cores and the gradual filling of surface pores. Subsequently, as the surface of the particles became smoother, the increase in pellet size accelerated, resulting in an S-shaped particle growth curve.

The developed imaging system enables the in-line monitoring of particle growth dynamics, thus allowing for a better process understanding. With the timely detection of out-of-spec products, the system allows for the adjustment of the critical process parameters, so that the final product meets the desired quality standards. For instance, higher than necessary inlet-air temperatures can cause the sprayed coating solution to dry too quickly, preventing proper adhesion to the particles and leading to reduced particle size and lower API content, which can be prevented through real-time monitoring and control. Additionally, the system can be used for endpoint detection, allowing the manufacturing process to be stopped once the batch meets the established specifications. This ensures consistent product quality and minimizes variability between batches, leading to more reliable manufacturing. Endpoint detection also facilitates energy efficiency by optimizing system runtime, thereby minimizing unnecessary energy expenditure.

In this study, the real-time particle size monitoring capability of the system was utilised; however, feedback control was not implemented. Although the real-time particle size data was not applied for feedback in this study, the availability of such data remains highly valuable for pellet coating processes. Real-time particle size information allows operators to shut down the process as soon as the particle size—or, more critically, the coating thickness—reaches the desired value. This represents a significant advantage, as traditional sampling and off-line measurements are time-consuming, often resulting in prolonged equipment operation due to the absence of reliable endpoint detection. Nevertheless, future studies should focus on utilising real-time particle size information for implementing feedback control to further optimise the process.

## 4 Conclusion

This paper presents a novel machine vision-based method for real-time particle size monitoring using an endoscopic imaging system. By capturing high-quality images of particles and employing a CNN model for instance segmentation, the system is able to accurately determine particle size and shape. The system was tested both off-line during particle fluidization and in-line during the pellet layering process. Comparison with reference measurements, including laser diffraction and dynamic image analysis, confirmed the reliability of the obtained particle size data. The imaging system, integrated in-line, successfully detected the increase in particle size during the pellet layering process, the results were consistent with off-line methods. The shape analysis results aligned with those from reference measurements, demonstrating the system's ability to accurately characterize particle morphology. The developed system offered valuable insight into the dynamics of particle size increase during the pellet layering process.

The endoscopic imaging system proved to be a feasible PAT tool, enhancing process understanding and improving quality control. Real-time measurement of critical quality attributes enables the timely detection of out-of-spec products, allowing for adjustments to critical process parameters and improving process control and efficacy. Employing in-line particle size monitoring tools for endpoint detection ensures the manufacture of products with consistent quality. Additionally, endpoint detection contributes to energy efficiency by ensuring the system is stopped when the process is complete, thereby avoiding unnecessary energy consumption and operational wear. Future research could integrate the detection of agglomerates into the CNN model with further training, allowing the system to detect additional types of product failure. As a continuation of the research, the imaging system could be tested in the case of different pellet-related processes, such as pelletization (layering processes) and filmcoating of pellets.

## CRediT authorship contribution statement

**Orsolya Péterfi:** Writing – original draft, Visualization, Software, Methodology, Investigation, Data curation. **Nikolett Kállai-Szabó:** Writing – review & editing, Supervision, Resources. **Kincső Renáta Demeter:** Methodology, Investigation. **Ádám Tibor Barna:** Resources, Investigation. **István Antal:** Writing – review & editing, Resources. **Edina Szabó:** Investigation. **Emese Sipos:** Writing – original draft, Supervision. **Zsombor Kristóf Nagy:** Writing – review & editing, Supervision, Project administration, Conceptualization. **Dorián László Galata:** Writing – review & editing, Supervision, Investigation.

## Declaration of competing interest

The authors declare that there are no conflicts of interest.
