# Supplementary material for: Artificial intelligence-aided endoscopic in-line particle size analysis during the pellet layering process
Source: J Pharm Anal. 2025 Feb 12;15(8):101227. doi: 10.1016/j.jpha.2025.101227 (PMC12491717; doi:10.1016/j.jpha.2025.101227)
Supplement: Multimedia component 1 [file mmc1.docx]

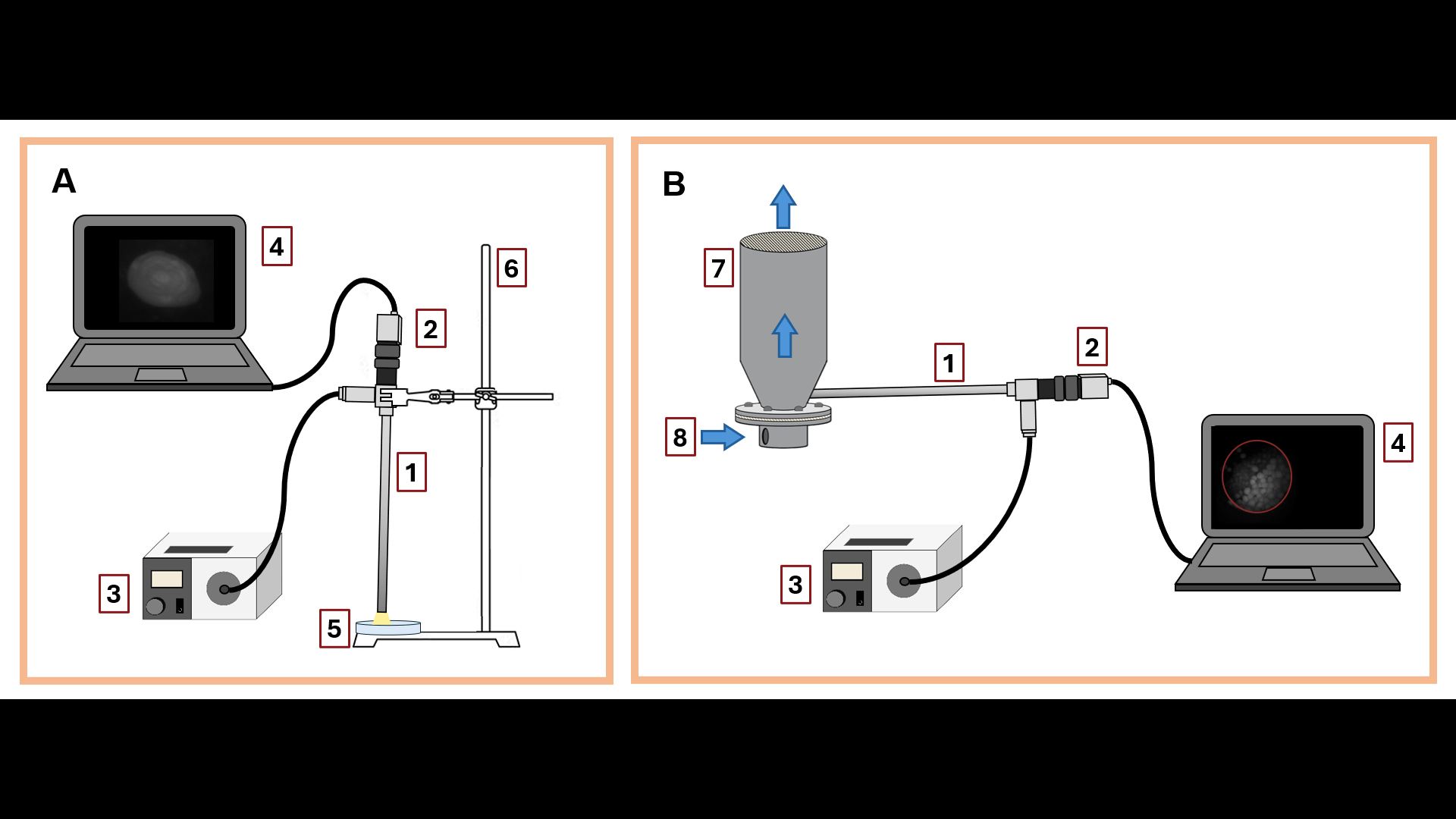


***Fig. S1.*** Experimental setup for off-line particle size analysis: (A) single particle analysis, (B) off-line analysis during particle fluidization. The key components of the system are the endoscope (1), camera (2), light source (3), and computer (4). For single pellet analysis, the sample (5) was placed under the endoscope, which was secured by a laboratory stand (6). For the PSD measurement of larger sample sizes, pellets were placed in a custom 3D-printed device (7), with fluidization achieved using pressurized air (8).


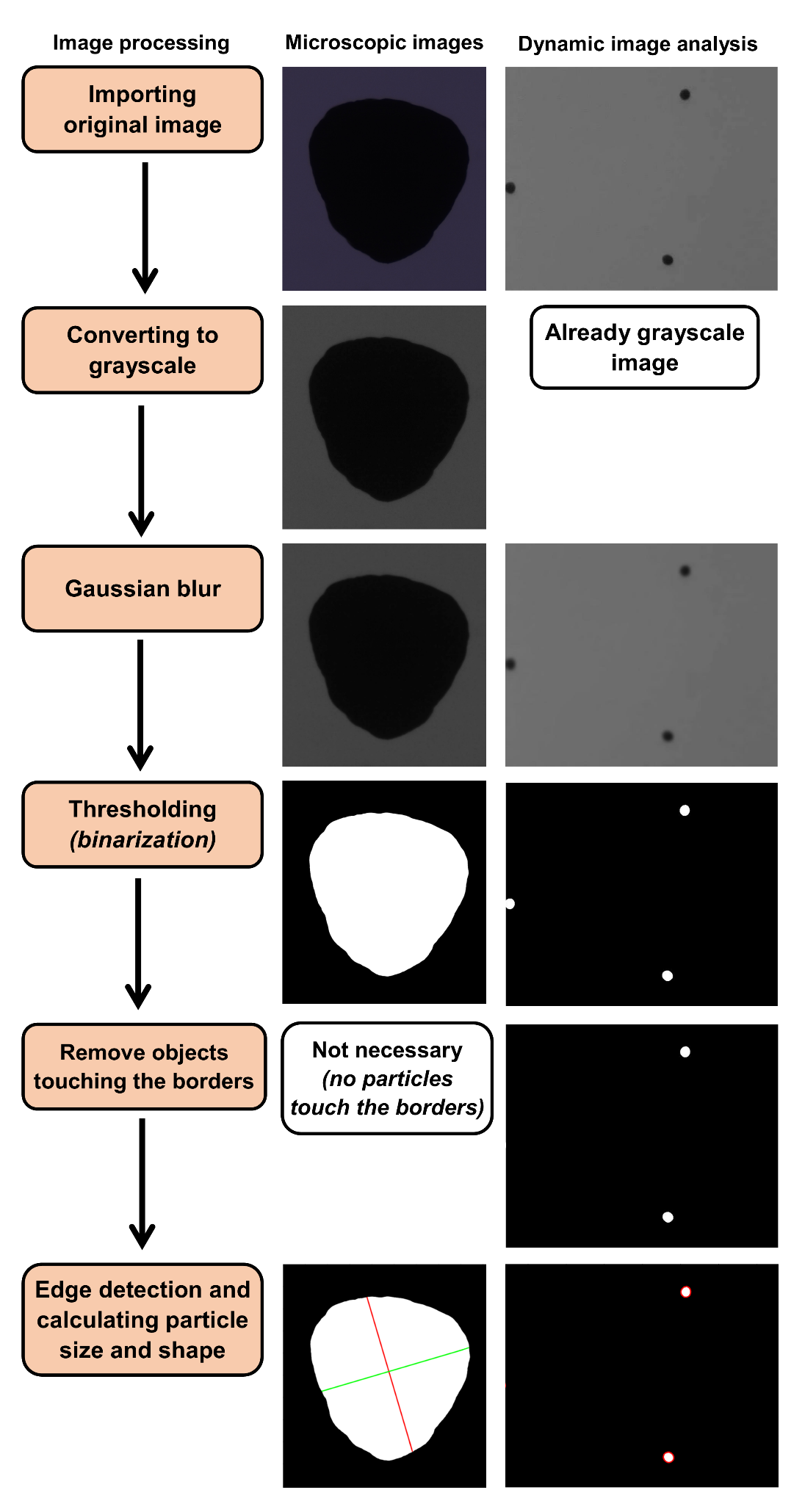


***Fig. S2.*** The main image processing steps in the case of the microscopic image and off-line dynamic image analysis.


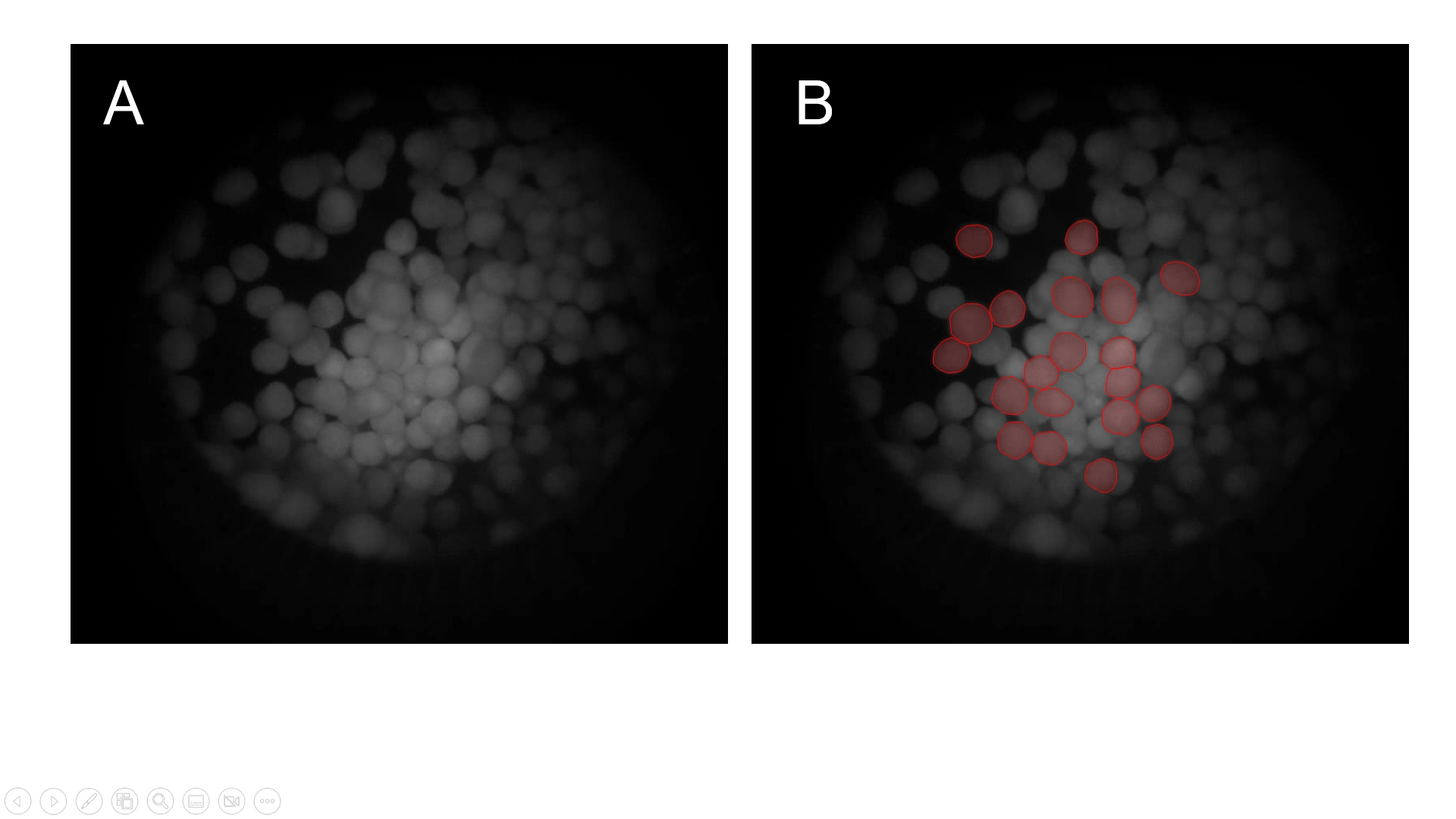


***Fig. S3.*** Particle detection using the trained CNN model for MCC pellet cores.

***Table S1.*** The mean aspect ratio and circularity of the pellets measured with the AI-based endoscopic system and off-line dynamic image analysis.

| Mass of added binder solution (g) | Endoscopic imaging system | | Off-line dynamic image analysis | |
| --- | --- | --- | --- | --- |
|  | Mean aspect ratio  ± SD | Mean circularity ± SD | Mean aspect ratio  ± SD | Mean circularity  ± SD |
| 0g | 0.870 ± 0.070 | 0.956 ± 0.031 | 0.851 ± 0.080 | 0.962 ± 0.026 |
| 50g | 0.874 ± 0.072 | 0.958 ± 0.029 | 0.853 ± 0.078 | 0.961 ± 0.025 |
| 100g | 0.872 ± 0.071 | 0.956 ± 0.032 | 0.855 ± 0.081 | 0.963 ± 0.027 |
| 200g | 0.873 ± 0.069 | 0.957 ± 0.032 | 0.854 ± 0.081 | 0.964 ± 0.026 |
| 300g | 0.871 ± 0.075 | 0.956 ± 0.034 | 0.855 ± 0.089 | 0.963 ± 0.032 |
| 400g | 0.878 ± 0.068 | 0.963 ± 0.029 | 0.857 ± 0.085 | 0.965 ± 0.027 |
| 500g | 0.876 ± 0.073 | 0.961 ± 0.029 | 0.856 ± 0.082 | 0.965 ± 0.028 |
| 600g | 0.880 ± 0.069 | 0.964 ± 0.027 | 0.858 ± 0.078 | 0.967 ± 0.025 |
